# Pregnant maternal brain dorsal anterior cingulate cortex choline/creatine ratios on 1H-MR spectroscopy in opioid exposure

**DOI:** 10.3389/fnins.2025.1569558

**Published:** 2025-04-16

**Authors:** Jonathan A. Class, Ramana V. Vishnubhotla, Yi Zhao, Nathan Ooms, David M. Haas, Senthilkumar Sadhasivam, Rupa Radhakrishnan

**Affiliations:** ^1^Department of Radiology and Imaging Sciences, Indiana University School of Medicine, Indianapolis, IN, United States; ^2^Department of Biostatistics and Health Data Science, Indiana University School of Medicine, Indianapolis, IN, United States; ^3^College of Health and Human Sciences, Purdue University, West Lafayette, IN, United States; ^4^Department of Obstetrics and Gynecology, Indiana University School of Medicine, Indianapolis, IN, United States; ^5^Department of Anesthesiology and Perioperative Medicine, University of Pittsburgh School of Medicine, Pittsburgh, PA, United States

**Keywords:** MR spectroscopy, pregnancy, opioid use disorder, prenatal opioid exposure, anterior cingulate cortex

## Abstract

There is growing interest in understanding the effects of opioid use on the brain, yet the effects of opioid use on the pregnant maternal brain are still relatively unknown. Pregnant women with opioid exposure during pregnancy are at high risk for adverse neurological and neuropsychiatric outcomes. Much of what is currently known about the impact of opioids on the maternal brain is mainly derived from studies in animal models; however, species-specific opioid pathways and other socio-environmental factors complicate the interpretation of results. A few studies in non-pregnant adults have shown the utility of magnetic resonance spectroscopy (MRS) in risk prediction in substance exposure. We know that pregnancy alters the pharmacodynamics and pharmacokinetics of opioid metabolism, and the impact of opioids on synapses may differ during pregnancy compared to the non-pregnant state. We, therefore, aimed to understand the neurometabolic alterations in pregnant women on medications for opioid use disorder (MOUD). In our multicenter study, we utilized 1H MRS to analyze metabolic alterations in the dorsal anterior cingulate cortex (dACC) in pregnant women on MOUD (12 subjects) vs. pregnant control women (21 subjects) without substance exposure. Using multivariable linear regression, we identified a positive association between opioid exposure and choline-to-creatine (Cho/Cr) ratios after correcting for gestational age and scanner site. We also identified a significant elevation in the Cho/Cr ratio in pregnant women on MOUD and concomitant polysubstance exposure when compared to pregnant women on MOUD without exposure to other substances and control pregnant women. These altered metabolite concentrations that we identified in the dACC may provide a mechanistic understanding of the neurobiology of MOUD and insights for better management and outcomes.

## Introduction

Opioid use in pregnancy has been reported as 12% in a large population-based cohort study and as high as 21% in Medicaid-enrolled women (Desai et al., 2014; Nechuta et al., 2022). From 2017 to 2020, opioid use in pregnancy was associated with higher maternal mortality (Bruzelius and Martins, 2022), especially with fentanyl, rather than prescription opioids or heroin.

Opioid use disorder (OUD) in pregnancy is associated with several comorbidities, such as polysubstance use and mental health disorders, which may be superimposed on the psychological changes in pregnancy, including mood disturbances, stress, and anxiety (Bjelica et al., 2018). A study found that 89% of US women of reproductive age with non-medical opioid use reported additional substance use (Jarlenski et al., 2017). In a retrospective primary care study of over 7,000 women, ~86% of women with opioid use disorder had comorbid mental health disorders (Braciszewski et al., 2022). Depression, anxiety, attention-deficit/hyperactivity disorder (ADHD), and post-traumatic stress disorder (PTSD) were common mental health conditions (Braciszewski et al., 2022; Huhn and Dunn, 2020) in pregnant women with opioid use disorder. Comprehensive care, in addition to medications for opioid use disorder (MOUD), is essential for these women (Center for Substance Abuse Treatment, 2005).

Methadone and buprenorphine are opioid medications and first-line therapy options for pregnant women with OUD (Center for Substance Abuse Treatment, 2005; Committee of Obstetric Practice, 2017). These medications reduce cravings and subsequent consequences such as withdrawal, relapse, overdose, lack of prenatal care, and preterm birth (Committee of Obstetric Practice, 2017; Suarez et al., 2022; Winklbaur et al., 2008). These benefits outweigh the small potential risks of such opioid therapy impacting brain plasticity (Thompson et al., 2021; Upadhyay et al., 2010).

Altered brain structure and functional connectivity have been demonstrated in the setting of opioid use. Opioids have been shown to induce dendritic changes in the nucleus accumbens and decrease dendritic spine density (Liao et al., 2007, 2005; Thompson et al., 2021). Additionally, significant decreases in functional connectivity were observed in regions including the amygdala and nucleus accumbens (Upadhyay et al., 2010). Since opioids predominantly affect neuronal function through their actions on opioid receptors and neurotransmitter release, understanding the underlying neuro-metabolite changes in the brain is crucial for getting a comprehensive picture of their impact on the brain. MR spectroscopy (MRS) is a magnetic resonance modality that captures certain metabolite levels in a small, predefined volume. MRS has provided insight into neuro-metabolite changes in the setting of opioid use and concurrent comorbidities such as polysubstance use and mental health disorders.

Opioid exposure, both illicit use and opioid maintenance therapy, may alter brain metabolite levels in several brain regions as studied in non-pregnant populations (Greenwald et al., 2015; Hermann et al., 2012; Murray et al., 2016; Yücel et al., 2007), with the anterior cingulate cortex being the most consistently affected. Glutamate has been a widely studied metabolite in addiction (Gass and Olive, 2008). Glutamate, a major excitatory neurotransmitter and mediator of synapse plasticity, may be chronically destabilized in addiction and contribute to relapse (Gass and Olive, 2008; Kalivas, 2009). This may be reflected as decreased glutamate in the setting of substance use (Yücel et al., 2007; Hermann et al., 2012). The glutamatergic system is also shown to prompt the rewarding effects of opioids that form opioid memories (Heinsbroek et al., 2021). In addition to glutamate, elevated brain choline is also suggested as a sign of synaptic adaptation to substance exposure (Hermann et al., 2012; Upadhyay et al., 2010). Other substances, such as cocaine and marijuana, have also been linked to metabolite changes in the anterior cingulate cortex in non-pregnant subjects (Newman et al., 2020; Prescot et al., 2013; Yang et al., 2009). Along with substance use, brain metabolite levels have been investigated in mental health disorders, a known comorbidity in pregnant women with opioid exposure.

Current literature on MRS in the setting of mental health disorders shows a mixed picture. For example, the association between depression and brain choline levels has been variable (Riley and Renshaw, 2018), and overall brain metabolic changes in MRS in ADHD have been inconsistent (Firouzabadi et al., 2022). However, choline levels in the anterior cingulate cortex (ACC) tend to be increased in PTSD, bipolar disorder, and ADHD, though few mechanistic assertions are made (Colla et al., 2008; Kong et al., 2023; Perlov et al., 2007; Scotti-Muzzi et al., 2021; Swanberg et al., 2022). Additionally, to our knowledge, these associations have not been studied in a pregnant human population.

Brain metabolite levels in pregnancy have been studied to a limited extent (McEwen et al., 2021; Rutherford et al., 2003). One study found a decrease in total choline in pregnancy 2–3 weeks prior to delivery compared to non-pregnant women (McEwen et al., 2021). To our knowledge, brain metabolite changes in substance use during pregnancy have not been studied. Since physiological changes in pregnancy can affect opioid pharmacokinetics and pharmacodynamics, extrapolating results from studies in non-pregnant adults to pregnant populations may be challenging.

Our study aimed to assess alterations in brain metabolite levels in the dorsal anterior cingulate cortex (dACC) in pregnant women on medication for opioid use disorder (MOUD) compared to healthy control pregnant women without opioid exposure, using H^1^MR spectroscopy. We hypothesized that on MRS, glutamate levels would be lower in women on MOUD, and choline levels would be elevated in women on MOUD when compared to control pregnant women. Our secondary goal was to assess the impact of polysubstance use and other mental health comorbidities on these brain metabolites.

## Materials and methods

### Subjects

Pregnant women >16 weeks of gestation were recruited as part of a prospective, IRB-approved, multisite study. The two study sites were Indiana University School of Medicine and the University of Pittsburgh Medical Center. Informed consent was obtained. Two groups of pregnant women were enrolled in this study: one group of pregnant women was prescribed MOUD, either buprenorphine or methadone, while the control group had no opioid exposure. Exclusion criteria included MRI contraindications, serious maternal medical illness, HIV/AIDS, and major fetal congenital abnormalities.

### Demographic and clinical data collection

Demographic and clinical data were obtained by patient interview and chart review and electronically stored on REDCap (Harris et al., 2019, 2009), hosted at each university site. Clinical data collected included details of substance use and mental health disorders. Pregnant women on MOUD were being followed by a physician. Tobacco use and polysubstance use were determined by a patient interview and urine drug screen. Polysubstance use included active use of any of the following substances: non-prescribed opioids, marijuana, cocaine, benzodiazepines, alcohol, and/or amphetamines. Maternal education was assessed through patient interviews. The presence of mental health disorders was extracted from chart reviews or through patient interviews. Depression was defined as either the subject currently being on medication for major depressive disorder (MDD) or scoring as moderate or severe on the Hamilton Depression Scale or Patient Health Questionnaire-9 (PHQ-9). Anxiety was defined as either the subject having a diagnosis of general anxiety disorder (GAD) or scoring as moderate or severe on the Hamilton Anxiety Scale or the General Anxiety Disorder-7 (GAD-7) scale. ADHD, bipolar disorder, and PTSD diagnoses were obtained from the medical history.

### 1H MR spectroscopy acquisition

**Figure 1:**
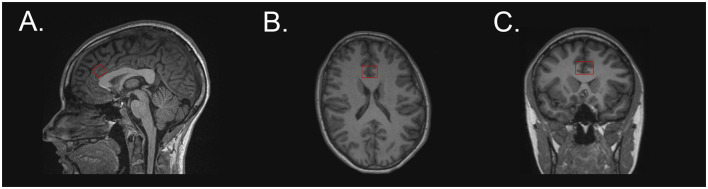
Voxel placement at the dorsal anterior cingulate cortex on a T1-weighted image in the following planes: **(A)** sagittal, **(B)** axial, and **(C)** coronal.

A brain MRI, which included anatomic scans and single-voxel 1H MR spectroscopy, was performed during the second or third trimester. All women at the Indiana University site underwent the same imaging protocol on a 3T Siemens Vida Fit scanner (Erlangen, Germany) with a 64-channel head coil. At the University of Pittsburgh site, enrolled women underwent the same imaging protocol on a 3T Siemens Skyra scanner (Erlangen, Germany). We utilized single-voxel point-resolved spectroscopy (PRESS) sequence with the following parameters: TR = 2,000, TE = 30, 128 averages. Spectra were obtained from the dorsal anterior cingulate cortex (dACC) (15 × 20 × 15 mm^3^) positioned above the genu/anterior body of the corpus callosum and tilted to match the curvature of the corpus callosum in the sagittal plane. Figure 1 is a depiction of this placement.

### Metabolite concentration quantification

**Figure 2:**
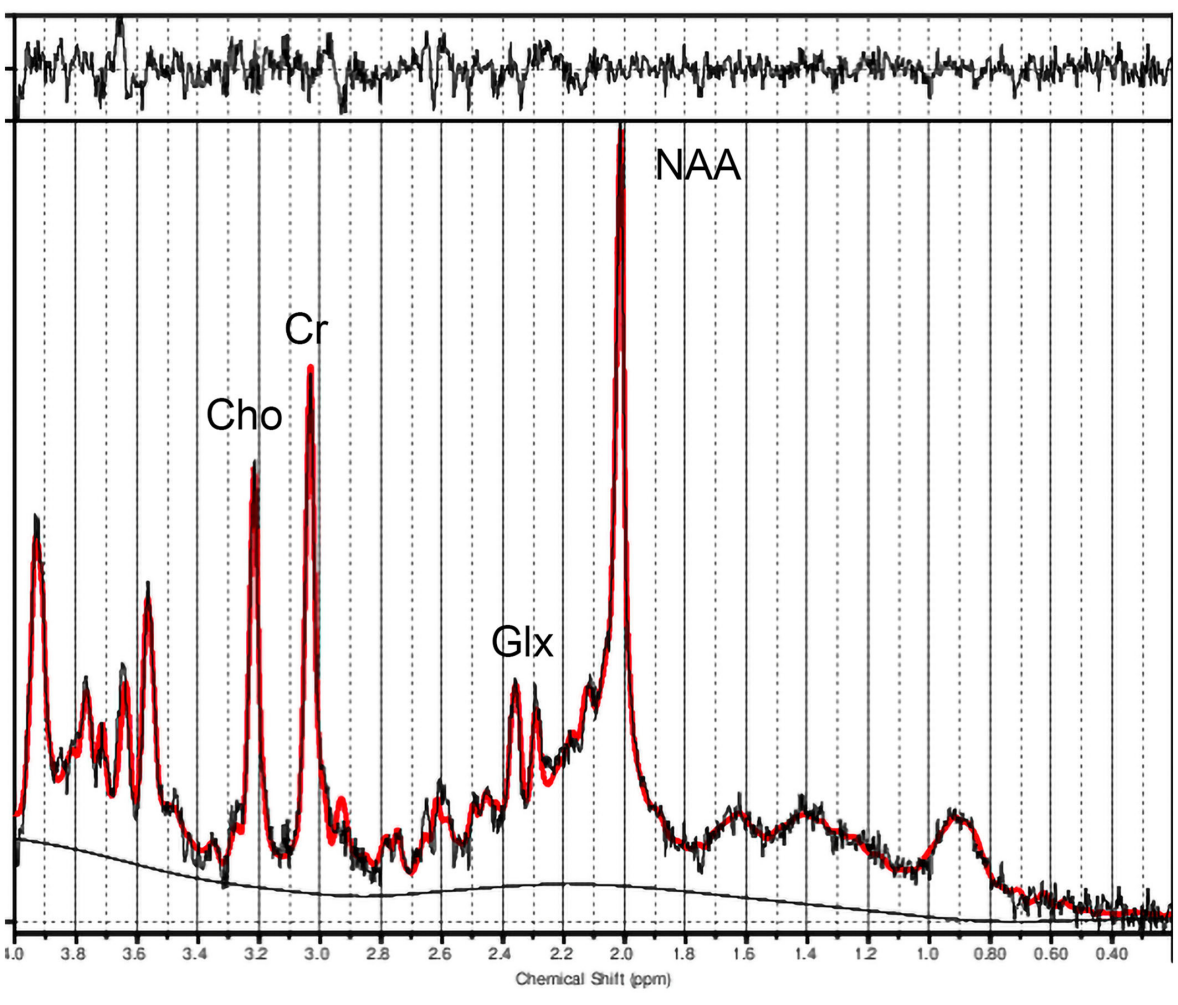
LCModel fitted spectrum from our study.

Metabolite concentration ratios were derived using LCModel software (Provencher, 2001). We utilized the LCModel basis spectra 3T, PRESS, TE30. We visually inspected the spectral quality, and spectral fits were accepted if CRLB was <20% (Hermann et al., 2012; Li et al., 2020), which was true for all spectra, with one spectrum displayed in Figure 2. The following metabolite concentrations were obtained with creatine plus phosphocreatine (Cr) as the denominator in the ratio: N-acetylaspartate and N-acetylaspartylglutamate (NAA + NAAG), choline (Cho) compounds, glutamate (Glu), glutamate + glutamine (Glx), and myo-inositol (Ins). For our study, we focused on Cho/Cr and Glx/Cr.

### Statistical analysis

Statistical analysis was performed in R (https://www.R-project.org/) (R Core Team, 2024), including Cohen's f2 (Cinelli et al., 2024) and ggplot2 for plots (Wickham, 2016).

Independent, multivariable regressions were performed with Cho/Cr and Glx/Cr as outcome variables. MOUD, gestational age, and scanner site were used as predictor variables, with MOUD and gestational age as variables of interest. We did not use maternal age in our analysis, as all our subjects were young adults in the reproductive age group. This age range is consistent with the age group (younger group cohort: 21–39 years old) chosen as a single age group in a previous AJNR study intended to study brain metabolite levels in normal aging (Angelie et al., 2001). Glx/Cr was chosen over Glu/Cr, given the overlap of the glutamate and glutamine peaks at 3T. Cohen's f2 was calculated to determine the partial effect size in our multivariable regression (Cohen, 1988; Selya et al., 2012). Metabolite ratios significantly associated with MOUD were evaluated using a robust linear regression comparing three groups: MOUD with polysubstance use (PSU), MOUD without PSU, and controls. We used gestational age and scanner site as covariates in the group analysis. We calculated Tukey's *p*-value for multiple testing corrections. A *p* < 0.05 was considered significant. We also calculated Cohen's *d* for effect size in the robust regression group comparison [t/square root (total N)]. Additionally, using the above covariates (MOUD, gestational age, and scanner site), we independently assessed the psychiatric comorbidities of depression, anxiety, ADHD, PTSD, and bipolar disorder. We performed a Bonferroni multiple comparison test for the psychiatric comorbidity *p*-values using R, which yielded adjusted *p*-values. These adjusted *p*-values were then compared to a significance level threshold of 0.05.

## Results

### Demographics and clinical characteristics

Demographics and clinical data are provided in Table 1. Subjects on MOUD showed exposure to other substances and tobacco. All mental health comorbidities except bipolar disorder were significantly greater in our MOUD group than in our control group. Supplementary Table 1 shows a further breakdown of clinical characteristics within the MOUD group. Tobacco use and mental health comorbidities were not significantly different between MOUD with PSU and MOUD without PSU.

**Table 1:** Demographic and clinical data.

	**MOUD**	**Control**	***p*-value**	
Number	12	21		
Demographic characteristics				
Age +/–SD (years)	28 +/−4.8	32.4 +/−4.8	0.015^*^	∧
Gestational age +/–SD (weeks)	29.8 +/−4.7	29.1 +/−4.4	0.26	∧
IU scanner site (%)	4 (33%)	9 (43%)	0.719	∧∧
Maternal education				
Incomplete high school	1 (8%)	1 (5%)		
HS diploma/GED	7 (58%)	8 (38%)		
Some college	4 (33%)	4 (19%)		
College graduate/advanced degree	0 (0%)	6 (29%)		
Unknown	0 (0%)	2 (10%)		
Polysubstance use (%)	7 (58%)	0 (0%)	1.85e-04^*^	∧∧
Tobacco use (%)	10 (83%)	0 (0%)	7.13e-07^*^	∧∧
Clinical characteristics				
Depression (%)	8 (67%)	5 (24%)	0.027^*^	∧∧
Anxiety (%)	10 (83%)	7 (33%)	0.01^*^	∧∧
Bipolar disorder (%)	4 (33%)	2 (10%)	0.159	∧∧
PTSD	7 (58%)	3 (14%)	0.016^*^	∧∧
ADHD	6 (50%)	1 (5%)	0.005^*^	∧∧

^*^Denotes significance.

∧Independent t-test.

∧∧Fisher's exact test.

### MOUD and Cho/Cr

**Figure 3:**
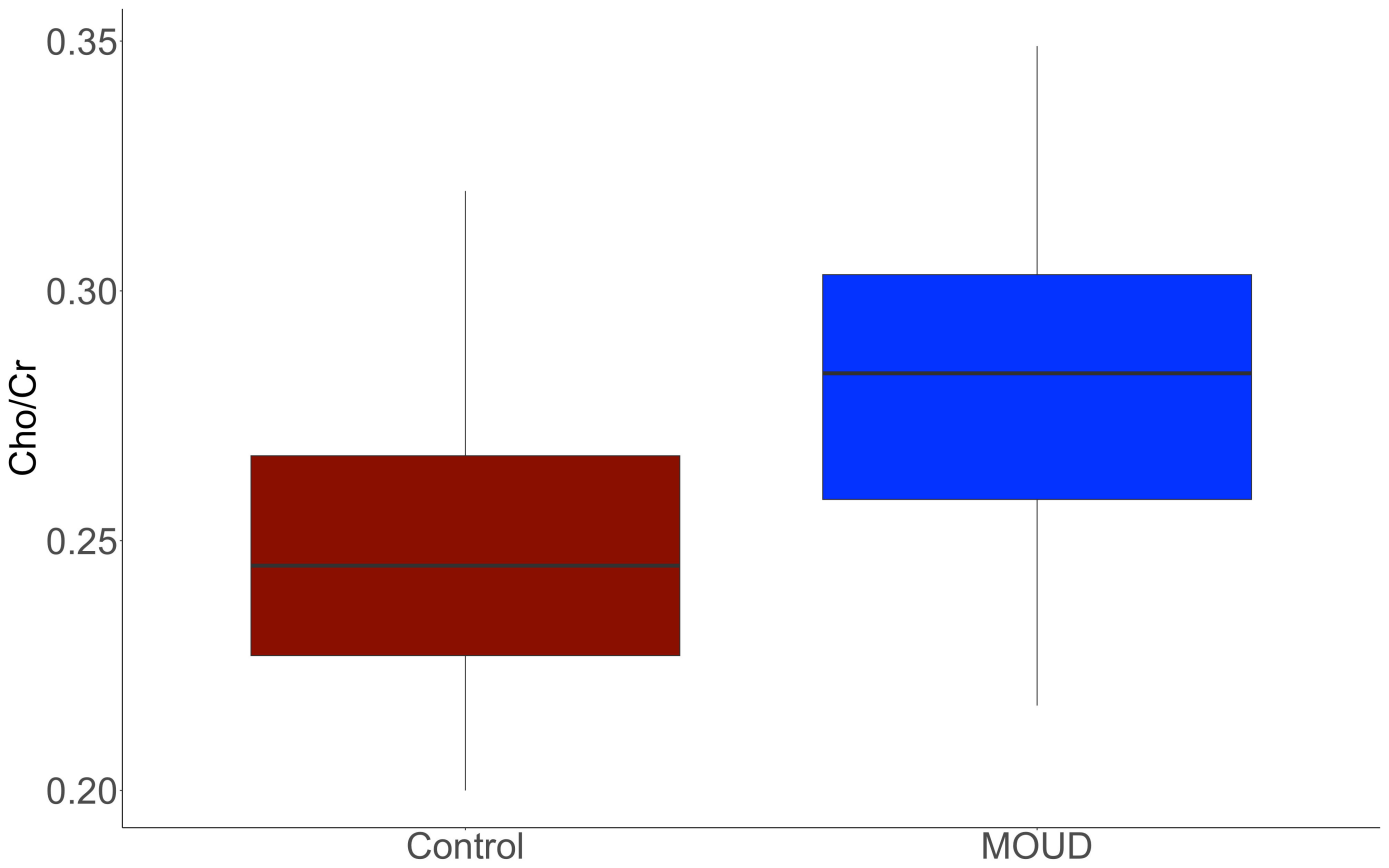
Cho/Cr boxplots of the MOUD and control groups.

**Figure 4:**
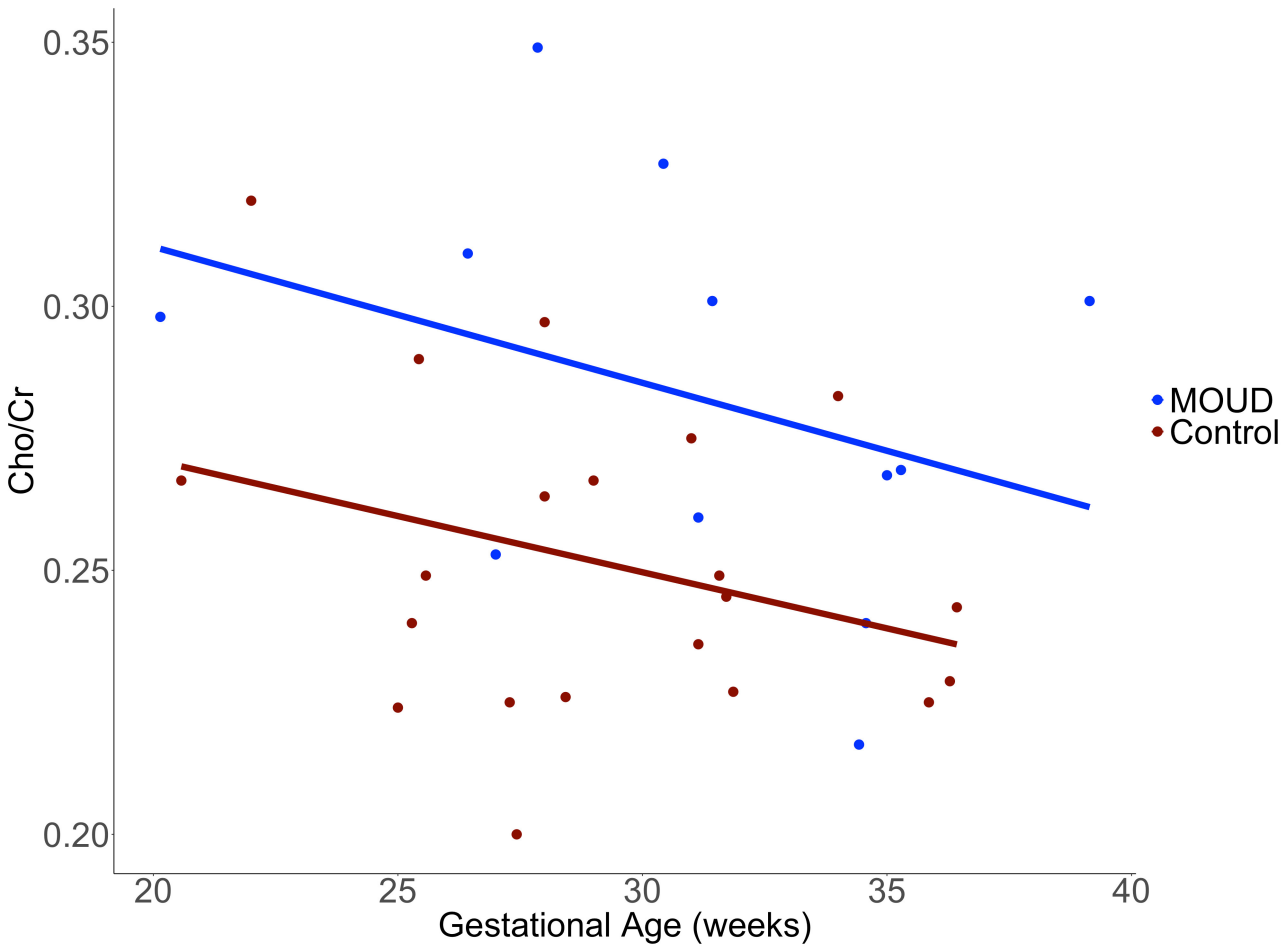
Scatterplot with regression lines showing the difference in Cho/Cr vs. gestational age (weeks) in our MOUD and control groups.

MOUD was positively associated with Cho/Cr when controlling for gestational age and scanner site (*t* = 2.96, *p* = 0.006). This result should be interpreted as opioid exposure in the context of our study population (concurrent tobacco use, polysubstance use, and mental health comorbidities). The Cohen's f2 partial effect size was 0.302, indicating a medium effect. Boxplots of MOUD and controls are displayed in Figure 3. Better seen in Figure 4, gestational age in both groups was negatively correlated with Cho/Cr, although this was not statistically significant (*t* = −1.87, *p* = 0.072).

### MOUD and Glx/Cr

Although Glx/Cr ratios in the dACC were lower in pregnant women on MOUD compared to control pregnant women when controlling for gestational age and scanner site (t = −1.495, *p* = 0.146, Cohen's f2 = 0.077, small partial effect), this association did not reach our threshold of statistical significance.

### Group differences

**Figure 5:**
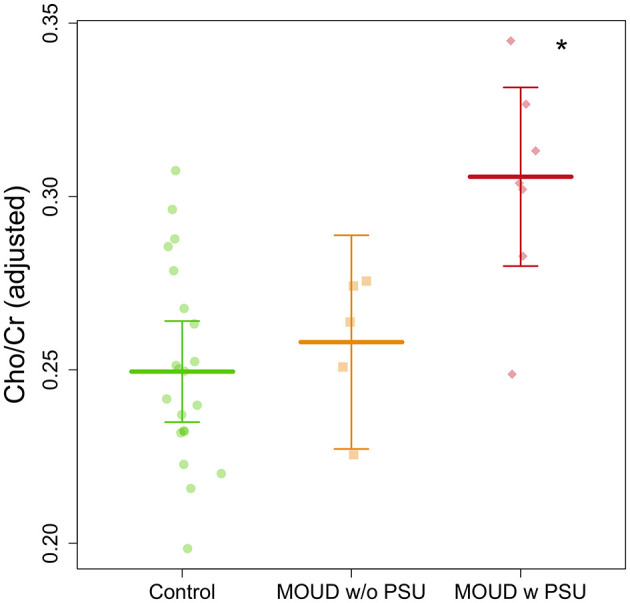
Group comparison of Cho/Cr ratios in pregnant women with MOUD with and without polysubstance use and controls after adjusting for gestational age and scanner site. MOUD with PSU is significantly greater (denoted by the *) than the other two groups.

Cho/Cr ratio on the dACC was significantly higher in the MOUD with polysubstance use (PSU) group (*n* = 7) compared to both the MOUD without PSU group (*n* = 5) and control group (*n* = 21) (*t* = 2.391, Tukey's *p* = 0.043; t = 3.861, Tukey's *p* = 0.0004, respectively) (Figure 5). Gestational age and scanner site were controlled in this group comparison. Cohen's *d* effect sizes were 0.730 for the MOUD with PSU to control comparison and 0.690 for MOUD with PSU to MOUD without PSU comparison, indicating medium effect sizes. There was no significant difference between the MOUD without PSU group and the control group (*t* = 0.507, Tukey's *p* = 0.865). Data are displayed in Figure 5.

### Mental health comorbidities

Partial correlations were independently calculated for the mental health disorders depression, anxiety, ADHD, bipolar disorder, and PTSD after accounting for scanner site, gestational age, and MOUD. ADHD was significantly associated with dACC Cho/Cr ratios (*t* = 3.398, *p* = 0.002), with a partial effect size of 0.412 (large effect size). Bipolar disorder was also significantly associated with Cho/Cr ratios (*t* = 2.837, *p* = 0.008), with a partial effect size of 0.287 (medium effect). The statistical significance of these associations survived multiple comparison tests. However, depression, anxiety, and PTSD were not significantly associated with Cho/Cr in the dACC in our analysis.

## Discussion

Pregnancy is accompanied by multiple physiological changes. Our study is the first MR spectroscopy study to analyze neuro-metabolite changes in substance exposure in the pregnant maternal brain. We identified a higher choline-to-creatine ratio on H1 MRS in the dorsal anterior cingulate cortex (dACC) in pregnant women on MOUD than in control pregnant women, particularly in the setting of PSU. Our results can help guide future studies and improve our understanding of the effects of substance use on the pregnant maternal brain and its effect on pregnancy outcomes.

Choline measured by MR spectroscopy is a combination of glycerophosphocholine, phosphocholine, and a small amount of free choline. Classically, total choline measured by MR spectroscopy represents membrane synthesis and degradation. This is most commonly clinically relevant for increased choline in the setting of tumors, representing rapid cell turnover (Zhu and Barker, 2011). Our study showed a significant positive association between Cho/Cr and MOUD, which, we hypothesize, may be a sign of adaptation and altered synapses (Hermann et al., 2012; Upadhyay et al., 2010). It is important to recognize, though, that this result may be driven by concurrent polysubstance use and psychiatric comorbidities, especially since the presence of PSU was also associated with higher Cho/Cr levels.

We identified that in pregnant women on MOUD, PSU was significantly associated with higher adjusted Cho/Cr levels in the dACC than in pregnant women on MOUD without PSU and controls. There was no significant difference in the Cho/Cr levels in pregnant women with MOUD without PSU and controls. Of note, tobacco exposure and mental health comorbidities were not significantly different between MOUD with PSU and MOUD without PSU, as shown in Supplementary Table 1. Overall, our results suggest that MOUD combined with concomitant polysubstance use may have a greater impact on neuro-metabolite concentrations and suggested synaptic changes, and impact future management. MOUD alone may not significantly alter Cho/Cr levels. We also investigated another metabolite of interest, glutamate.

Our study did not find an association between MOUD and Glx/Cr. Glx is a combination of glutamate and glutamine, with a strong correlation to glutamate. Glutamate pathways are thought to be impacted in addiction (Kalivas, 2009), yet studies assessing the association between Glx and OUD report mixed results. A negative association between Glx and opioid use has been previously reported in the literature (Yücel et al., 2007), but another study only found this trend in individuals below the age of 34 years (Hermann et al., 2012). Our results may differ from the first study due to the impact of pregnancy, our limited sample size, or associated comorbidities. As we had a negative trend with a small partial effect size, a larger sample size may have shown a negative association between MOUD and Glx. In the studies mentioned above and another opioid study (Liu et al., 2017), the ACC has been a region of interest.

The ACC is an important region in addiction-related neural networks (Zhao et al., 2020). Additionally, the ACC plays an important part in human maternal behavior neurocircuits, which help regulate parental behavior, including stress and anxiety responses (Swain and Ho, 2023). The ACC is also a region of interest in relation to the mental health comorbidities, which are more frequently encountered in pregnant women on MOUD. In our cohort, there was a positive association between mental health comorbidities (ADHD and bipolar disorder) and dACC Cho/Cr ratios, although these associations should be interpreted in the context of our small sample sizes. However, other studies on non-pregnant individuals have also previously reported increased Cho/Cr levels in the ACC in ADHD subjects compared to controls, hypothesized to be due to altered plasticity (Colla et al., 2008; Perlov et al., 2007). Similarly, this association of altered Cho/Cr ratios in the ACC has also been reported in non-pregnant individuals with bipolar disorder (Kong et al., 2023).

Another interesting relationship in our analysis was between gestational age and Cho/Cr ratios in the dACC. The negative trend we identified between gestational age and Cho/Cr could potentially be related to the increasing fetal choline demand throughout gestation (Jaiswal et al., 2023). This suggests a potential inverse relationship between fetal choline need and maternal choline levels, as proposed in a previous MR spectroscopy study comparing pregnant women to non-pregnant controls (McEwen et al., 2021). There is also some evidence that brain choline increases with increased dietary choline intake (Stoll et al., 1995), which may further suggest a link between systemic choline levels influencing brain choline levels.

Our study had several limitations. First, the sample size was limited, which reduces the power of our analysis. Although we used robust linear regression to help address the skewed sample size in our group comparison, the sample sizes within the MOUD subgroups were still small. While our results suggest directionality to alterations in brain metabolites and the presence of polysubstance use, these should be interpreted with prudence and be further studied in larger cohorts. Multicenter MRS imaging can introduce variability in metabolite concentrations (Považan, 2020). We controlled for the subject site, and the vendor was the same between the sites. We did not have unsuppressed water peaks available when creating spectra in the LCModel; therefore, we were unable to obtain absolute metabolite concentrations and relied on ratios with creatinine as our normalization. As noted above, many previous studies report these ratios. Although all women enrolled were in the childbearing age group, women on MOUD were on average younger (28 yrs +/−4.8) compared to the control group (32yrs +/−4.8). Previous studies have considered this age range as a single group (Angelie et al., 2001). However, older maternal age is shown to be associated with differences in infant brain development. The presence of multiple comorbidities, such as polysubstance use and mental health disorders, is a limitation to drawing concrete mechanistic conclusions in this study. To avoid overfitting and multicollinearity in our model, we did not include these comorbidities in our initial regressions. Tobacco exposure had a significant correlation with opioid exposure, so inferences of opioid exposure may be limited because of this and should be seen in the setting of concurrent tobacco use. As previously mentioned, these comorbidities are common to pregnant women with OUD, so our subjects are characteristic of the population we are studying. Furthermore, MOUD with PSU and MOUD without PSU were similar in terms of tobacco and mental health comorbidities. We did not account for the number of prior pregnancies in our analysis.

In addition to the dACC, we attempted to acquire spectra from the nucleus accumbens (NAcc), a small region (~1 cm^3^–2 cm^3^), therefore voxel placement was difficult in this region and inevitably included surrounding structures to achieve a large enough signal-to-noise ratio (Neto et al., 2008; Steinegger et al., 2021). Although efforts have been made to improve MR spectroscopy precision for the NAcc (Engeli et al., 2021; Steinegger et al., 2021), this adds a substantial amount of scan time. Given that these difficulties substantially affected our sample size, we did not include this region in our analysis.

Despite some of the abovementioned limitations, our study provides novel evidence of the potential impact of substance use on the pregnant maternal brain in women on MOUD. The ACC has been shown to play a role in regulating human maternal behavior, stress, anxiety, and maternal–infant bonding. Although we did not explore the direct clinical significance of the changes noted in the Cho/Cr ratio in the ACC in our study, we envision future research assessing this altered metabolic profile in the context of treatment adequacy in curbing craving, withdrawal, postpartum relapse, infant NOWs, and maternal–infant bonding.

## Conclusion

In this study, we identified alterations in choline-to-creatine ratios on H1 MR spectroscopy of the dorsal anterior cingulate cortex in pregnant women on medication for opioid use disorder compared to control pregnant women without opioid exposure. We also identified higher Cho/Cr ratios in pregnant women with MOUD and polysubstance use compared to pregnant women on MOUD without polysubstance use and control pregnant women. Mental health comorbidities—ADHD and bipolar disorder—were also associated with altered Cho/Cr ratios. This is the first study analyzing neuro-metabolite levels and substance exposure in a pregnant population, and our results strengthen the importance of addressing maternal comorbidities in these women. Our results can help direct future research in brain metabolite alterations to understand underlying mechanisms, predict and improve outcomes, and create personalized treatments for women on MOUD and with associated comorbidities.

## Data Availability

The raw data supporting the conclusions of this article will be made available by the authors, without undue reservation.
