# Supplementary material for: Pregnant maternal brain dorsal anterior cingulate cortex choline/creatine ratios on 1H-MR spectroscopy in opioid exposure
Source: Front Neurosci. 2025 Apr 16;19:1569558. doi: 10.3389/fnins.2025.1569558 (PMC12040935; doi:10.3389/fnins.2025.1569558)
Supplement: Supplementary file 1 [file Table_1.docx]

|  | MOUD w/ PSU | MOUD w/o PSU | p-value^ |
| --- | --- | --- | --- |
| Number | 7 | 5 |  |
| Demographic and Clinical Characteristics |  |  |  |
| Maternal education (≤ HS diploma) | 4 | 4 | 0.576 |
| Tobacco exposure (%) | 6 (86%) | 4 (80%) | 1 |
| Depression (%) | 6 (86%) | 2 (40%) | 0.222 |
| Anxiety (%) | 7 (100%) | 3 (60%) | 0.152 |
| Bipolar disorder (%) | 3 (43%) | 1 (20%) | 0.558 |
| PTSD (%) | 5 (71%) | 2 (40%) | 0.576 |
| ADHD (%) | 4 (57%) | 2 (40%) | 1 |
| ^p-values from Fisher's exact test |  |  |  |
|  |  |  |  |

Supplementary Table 1. Comorbidities for MOUD subgroups, MOUD w/ PSU and MOUD w/o PSU.
